# Benchmarking Artificial Neural Network Architectures for High-Performance Spiking Neural Networks

**DOI:** 10.3390/s24041329

**Published:** 2024-02-19

**Authors:** Riadul Islam, Patrick Majurski, Jun Kwon, Anurag Sharma, Sri Ranga Sai Krishna Tummala

**Affiliations:** Department of Computer Science and Electrical Engineering, University of Maryland, Baltimore County, Baltimore, MD 21250, USA; majurski@umbc.edu (P.M.); junkyuk1@umbc.edu (J.K.); alnu1@umbc.edu (A.S.); stummal1@umbc.edu (S.R.S.K.T.)

**Keywords:** artificial neural network, ANN, spiking neural network, SNN, convolutional neural network, CNN, ANN-to-SNN conversion, network-on-chip, NoC, low energy

## Abstract

Organizations managing high-performance computing systems face a multitude of challenges, including overarching concerns such as overall energy consumption, microprocessor clock frequency limitations, and the escalating costs associated with chip production. Evidently, processor speeds have plateaued over the last decade, persisting within the range of 2 GHz to 5 GHz. Scholars assert that brain-inspired computing holds substantial promise for mitigating these challenges. The spiking neural network (SNN) particularly stands out for its commendable power efficiency when juxtaposed with conventional design paradigms. Nevertheless, our scrutiny has brought to light several pivotal challenges impeding the seamless implementation of large-scale neural networks (NNs) on silicon. These challenges encompass the absence of automated tools, the need for multifaceted domain expertise, and the inadequacy of existing algorithms to efficiently partition and place extensive SNN computations onto hardware infrastructure. In this paper, we posit the development of an automated tool flow capable of transmuting any NN into an SNN. This undertaking involves the creation of a novel graph-partitioning algorithm designed to strategically place SNNs on a network-on-chip (NoC), thereby paving the way for future energy-efficient and high-performance computing paradigms. The presented methodology showcases its effectiveness by successfully transforming ANN architectures into SNNs with a marginal average error penalty of merely 2.65%. The proposed graph-partitioning algorithm enables a 14.22% decrease in inter-synaptic communication and an 87.58% reduction in intra-synaptic communication, on average, underscoring the effectiveness of the proposed algorithm in optimizing NN communication pathways. Compared to a baseline graph-partitioning algorithm, the proposed approach exhibits an average decrease of 79.74% in latency and a 14.67% reduction in energy consumption. Using existing NoC tools, the energy-latency product of SNN architectures is, on average, 82.71% lower than that of the baseline architectures.

## 1. Introduction

Spiking neural networks (SNNs) [1] represent the vanguard in the evolution of artificial neural networks (ANNs), drawing inspiration from the intricate workings of biological organisms. SNNs offer several advantages and unique features compared to traditional ANNs, including biological plausibility, making them more biologically plausible than ANNs. They operate using spikes, similar to the firing of neurons in the human brain. In addition, SNNs are inherently event-driven, meaning they process information only when there is a change (spike). This event-driven nature can lead to energy-efficient computations, especially in applications where continuous processing is not necessary. SNNs also naturally capture temporal information through the timing of spikes. This is essential for tasks where the sequence and timing of events matter, such as in sensory processing or dynamic pattern recognition. Most importantly, the sparsity and binary nature of spikes in SNNs can lead to energy-efficient hardware implementations. This is particularly advantageous for applications in edge computing and IoT devices, where power consumption is critical to enable real-time processing. In contrast to ANNs, SNNs can exhibit robustness to input noise, as their spike-based processing can filter out irrelevant information. This can be beneficial in applications where input data may have inherent noise.

However, the advanced neural network paradigm finds efficient implementation in neuromorphic platforms, which are characterized by manycore systems, wherein a predetermined quantity of neuronal computation is meticulously mapped to individual cores. Communication between these neurons, facilitated through synapses, is orchestrated using the network-on-chip (NoC) fabric—a quintessential design choice for engendering seamless communication within a multicore system. In the traditional SNN architecture, non-biological spiking neurons and grids, akin to the architecture of cache memory [1], are employed. The neuron undergoes a firing event, or spike production, immediately upon surpassing its action potential threshold, with the crossbars serving as repositories for synaptic weights [2].

The computational efficacy, gauged by execution latency and energy consumption, of an SNN-based computing system is contingent upon the judicious allocation of neurons to computing units (i.e., cores) with minimal communication latency. Nevertheless, the electrical constraints of the input load and output load impose limitations on the number of input–output connections per neuron, necessitating the incorporation of multiple crossbars through NoC architectures.

In this context, extant algorithmic methods for mapping SNN unitary computational components to cores in a manycore system need more consideration for the underlying NoC models to ensure the attainment of optimal communication delay. Furthermore, our investigation has identified multiple pivotal challenges in designing large-scale SNNs on actual hardware systems. These challenges include (i) a dearth of comprehensive guidelines for constructing a software-level model translating to hardware deployment, (ii) the absence of design-automation devices and the imperative need for a breadth of domain expertise, and (iii) limitations in existing neuron clustering approaches, which are incapable of handling a large number of neurons in an SNN. This research addresses the aforementioned challenges by offering an existing graph-partitioning algorithm [3] and effectuating the placement of SNN architectures onto an NoC model, employing a methodology of a generic nature.

In this manuscript, we address a significant limitation present in current graph-partitioning algorithms [1,4,5], specifically the constraint on the number of vertices, which typically remains below 10,000. We introduce our novel greedy graph-partitioning algorithm, which has the capacity to effectively manage graphs comprising over 100,000 vertices, thereby mitigating a substantial amount of communication overhead when integrated into crossbar hardware configurations [3]. In particular, the key contributions of this work are as follows:

- We introduce our novel design and automation methodology that systematically transforms any neural network architecture into an SNN for the purpose of optimizing energy efficiency in neuromorphic computations.
- We introduce our novel graph-partitioning algorithm devised for implementing extensive SNNs.
- We map partitioned SNN architectures to a state-of-the-art NoC tool flow to show the efficiency of the proposed methodology.
- We conduct benchmark assessments on diverse deep neural network (DNN) and convolutional neural network (CNN) architectures and seamlessly integrate multiple applications to demonstrate the efficacy of our tool flow.
- Compared to a baseline graph-partitioning algorithm, the proposed method showcases an average decrease of 79.74% in latency and a 14.67% reduction in energy consumption. Collectively, the proposed approach exhibits, on average, an 82.71% reduction in the energy-latency product compared to the baseline approaches.

## 2. Background

There has been a notable surge in research focused on SNNs in recent years [6,7,8,9]. The predominant catalyst for this increased attention is the energy-efficient operation characteristic of these networks, as highlighted in [10]. This aspect distinguishes SNNs from traditional low-power techniques, as documented in various studies [11,12,13,14,15,16,17,18,19,20,21]. SNN models are inherently reactive to event-based data, making them particularly apt for address-event representation-based computations, as explored in [22].

However, there are some key challenges in training SNNs, including the following: (i) **Non-differentiability** [23,24,25]: SNNs often use spiking activation functions, which are non-differentiable. Traditional gradient-based optimization techniques like backpropagation, widely used in training neural networks, face challenges in the non-differentiable spiking context. (ii) **Temporal Dynamics** [26,27]: SNNs operate on a temporal framework, where information is encoded in the timing of spikes. Training networks to effectively utilize temporal dynamics and learn precise spike timings can be complex. (iii) **Sparse and Binary Activations**: Spikes in SNNs result in sparse and binary activations, posing challenges in applying traditional optimization methods designed for continuous and dense activations. (iv) **Variable Spike Latency** [28]: The latencies of spikes can vary, introducing an additional temporal dimension to consider during training. Capturing and learning these variable spike latencies is a non-trivial task. (v) **Conversion from ANNs** [29]: Converting pre-trained ANNs to SNNs introduces challenges due to differences in their architectures and the spike’s temporal mismatch. (vi) **Lack of Standardization**: Unlike ANNs, which benefit from standardized architectures and practices, SNNs lack established standards. This can make it difficult to compare results across different studies and implementations. (vii) Hardware Limitations: Implementing and training large-scale SNNs on hardware platforms optimized for traditional neural networks can be inefficient. Specialized neuromorphic hardware is often required for efficient SNN training and inference. Addressing these challenges is crucial for advancing the field of SNNs and harnessing their potential in neuromorphic computing and other applications.

CARLsim, an interesting tool, is a robust C++ library extensively employed for training and simulating large, biologically detailed neural networks (NNs), as described in [10]. This simulator is proficient in leveraging multiple CPUs and GPUs simultaneously, facilitating heterogeneous computing platforms. In the realm of SNN development, an intriguing automation tool, the SNN Tool Box (SNN-TB), as introduced in [30], is noteworthy. This tool primarily utilizes an ANN to construct an SNN. A significant advantage of the SNN-TB is its capability to extract the SNN model for deployment in existing SNN simulators, as evidenced in [31]. Consequently, this tool was employed in our research for graph generation.

Graph partitioning is a critical process within the realm of electronic design automation (EDA). Within this context, a heuristic approach based on the Kernighan–Lin (KL) algorithm, as initially proposed by Kernighan and Lin in 1970 [32], is commonly employed for the bipartitioning of graphs. However, a notable limitation of existing graph-partitioning methodologies, as discussed in previous works [1,4,5], is their inability to efficiently process graphs with more than 10K vertices. The present study utilized our novel SNN graph-partitioning algorithm (SNN-GPA) to address this constraint [3]. This algorithm demonstrates a marked capability in handling graphs containing in excess of 100,000 vertices. Moreover, it significantly reduces the volume of communication required when these graphs are implemented within crossbar hardware architectures. The innovation of the SNN-GPA thereby represents a substantial advancement in the field of graph partitioning, particularly in the context of large-scale electronic design automation.

Artificial intelligence (AI) and machine learning (ML) are playing a key role in the advancement of future chip design, and the leading EDA companies (i.e., Cadence, Synopsys, etc.) are using AI/ ML to improve the productivity of their tools. Furthermore, researchers play a crucial role in the development of AI/ML-based EDA tools [33,34,35,36,37,38]. However, a generic end-to-end tool for ANN-to-SNN conversion and SNN-to-hardware layout is yet to be developed. We believe the proposed tool flow will advance this area by characterizing the conversion from ANN to SNN and mapping the SNN computation onto an NoC-based computing platform.

The importance of NoC [39,40] in manycore systems stems from the need to efficiently manage communication between many processing cores integrated on a single chip. As the number of cores on chips increases to improve computational power and efficiency, traditional bus-based and point-to-point communication architectures face significant scalability, bandwidth, and latency challenges. NoC is a critical solution to these challenges, offering several advantages that make it indispensable for manycore systems [41]. Previously, researchers have used NoC-based SNN implementations but with a limited number of neurons [1]. In addition, large-scale SNN implementations require a large number of neuronal activity computations, which is not feasible using a single-core machine. As a result, the use of an NoC-based manycore system is imperative for efficient SNN implementations.

## 3. Proposed Spiking Neural Network Implementation Tool Flow

### 3.1. Architectural Design of Spiking Neural Networks

**Figure 1:**
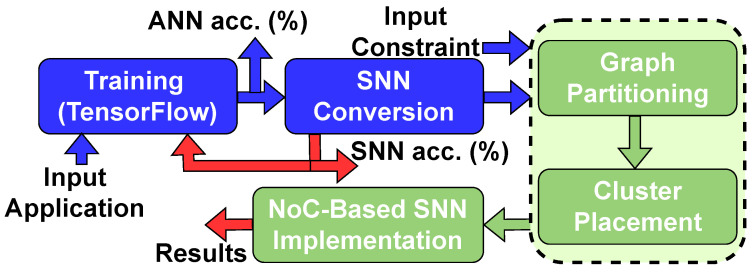
The proposed tool flow involves a sequence of structured processes. Initially, it trains an ANN model utilizing the TensorFlow framework. Subsequently, this trained model is converted into a spiking neural network (SNN). Thereafter, the tool flow applies the newly proposed graph-partitioning algorithm. This algorithm functions to efficiently cluster the neural network and facilitate its placement on an NoC grid, thus optimizing the network’s spatial distribution and operational efficiency [3].

**ANN Training:** In the scope of this paper, we introduce a sophisticated platform designed for the exploration of high-level neural architectures, focusing on the accurate characterization and implementation of SNNs. Initially, an NN model is developed using a well-established software library, namely Keras [42], and subsequently implemented on the TensorFlow platform [43]. Our proposed tool flow is shown in Figure 1 [3]. It is equipped to support a range of conventional Keras layers, including the following: A fully connected layer: Each neuron in the pre-synaptic group is connected to each neuron in the post-synaptic group. A 2D convolutional layer: For each stride of the kernel across the input group, synapses are created from each input neuron within the kernel to the respective output neuron. This is repeated for each filter in the convolutional kernel, creating connections between input and output neurons for each position of the kernel. When the kernel size is larger than the stride, input neurons are connected to multiple output neurons. A flattening layer: Flattened layers are not represented as neurons in the SNN. Instead, they serve to reshape the dimensions of the input layer to accommodate the shape of the following layer. Average pooling: Pooling layers have neurons and synapses in the output graph, preserving channels like the original CNN. Synapses for average pooling connections use alternate calculations for destination neuron activation [30]. A dropout layer: Dropout layers do not appear in the parsed SNN model, as they do not have weights or spike trains. A batch normalization layer: Normalization layers do not have weights or spike trains, and they typically follow an activation layer.

**ANN-to-SNN Conversion:** Following this, the NN model, once trained, is transformed into an SNN model using an existing SNN converter [30]. This conversion process involves two critical steps. In the first step, the ANN or CNN undergoes a transformation into an intermediate architecture. During this phase, dropout and batch normalization layers are either strategically eliminated or seamlessly integrated into the connected layers.

At this juncture in the process, the weights for each layer are subjected to normalization. Subsequently, the preprocessed CNN is further transformed into an SNN architecture. It is pertinent to note that the SNN converter is compatible with a variety of simulator backends, as documented in [31,44]. However, the built-in INI backend is employed in the current implementation, utilizing a temporal mean rate approximation to facilitate the simulation.

In the process of converting CNN weights to SNN weights, a methodology is employed that leverages the dynamics of neuronal membranes. Within this framework, the firing rate of spiking neurons is established to be proportional to the weights present in the CNN. This proportionality is a critical aspect of the conversion process, ensuring that the intrinsic characteristics of the CNN are preserved in the SNN representation. The weights and biases derived from the CNN are subjected to a normalization procedure to facilitate this conversion. This normalization is executed using a factor determined by the k-percentile of the total activity distribution within a given network layer. The primary objective of this normalization step is to mitigate approximation errors that may arise from excessively low or high neuronal firing rates. Such errors can significantly distort the representational fidelity of the SNN, hence the need for this precautionary measure. Furthermore, in the context of the SNN, the weights are permitted to assume both positive and negative values. This allowance is crucial as it enables the SNN to encapsulate a broader range of dynamics and interactions reflective of those present in the original CNN. This flexibility in the weight values plays a pivotal role in preserving the computational capabilities and characteristics of the CNN within the converted SNN framework.

In contrast to traditional approaches, this research entails validating the SNN model’s accuracy utilizing test data. Following this, the spike events of each neuron are extracted, along with the classification accuracy. The methodology allows for the use of either constant current or Poisson spike trains as the input current. Subsequently, our proposed tool flow employs the SNN model and neuron firing data to effectively decode the ANN interconnection between layers into corresponding synapses. The comprehensive tool flow of this process is depicted in Figure 1. During the development phase, an SNN graph is constructed, taking into consideration the firing rate of the pre-synaptic neuron and the weight of the synaptic connection.

### 3.2. Graph Partitioning and Cluster Placement

Upon completion of training an SNN, a connected graph is extracted from the model. The proposed graph-partitioning algorithm then clusters the neurons, taking into account the synaptic weights and the pair-wise layers within the SNN. This algorithm is grounded in the principles of the established Kernighan–Lin (KL) algorithm, as referenced in [32]. It meticulously evaluates both the intra-communication weights (IntraW)—pertaining to neurons residing within the same cluster—and the inter-communication weights (InterW)—associated with neurons located in distinct clusters. Such an approach ensures a comprehensive consideration of synaptic connections during the clustering process.

The novel SNN graph-partitioning algorithm (SNN-GPA), as delineated in Algorithm 1, presents a structured methodology for neural network partitioning [3]. The SNN-GPA accepts three key inputs: an SNN represented as a graph (*G*), the specified number of clusters (*N*), and the total number of layers (*n*). Its output comprises partitions or a collection of subgraphs. Notably, the number of clusters is determined by the constraint on the maximum permissible number of neurons per cluster.

Within its operational framework, the SNN-GPA calculates the number of clusters for each pair of layers and then proceeds to generate a set of initial random graphs or partitions, focusing on consecutive layers, as indicated in Line 4 and Line 5 of the algorithm, respectively. Subsequently, through an iterative process, each partition is converted into random clusters. The Kernighan–Lin method (KLmethod()) is then applied, as delineated from Line 6 to Line 11, with the objective of maximizing IntraW and minimizing InterW.
**Algorithm 1:** SNN graph-partitioning algorithm1:**Input:** G(V,E), vertices (*V*) and edges (*E*) of the graph; *N*, # of clusters; *n*, # of layers;2:**Output:** *P* partitions;3: 4:$N^{\prime }=N/\left(n-1\right)$     ▹ Calculate the aggregate number of clusters within a pair of layers.5:$G^{\prime }=\left\{G_{1}^{\prime },G_{2}^{\prime },...,G_{n}^{\prime }\right\}=RandGraph\left(G,n\right)$     ▹ Generate a randomized partition while considering successive layers.6:**for all** $G_{i}^{\prime }\in G^{\prime }$**do**:7:       $C=\left\{C1,C2,...,C_{N}\right\}=RandCut\left(G_{i}^{\prime },N^{\prime }\right)$     ▹ Generate randomized clusters from each of the initial partitions.8:    **for all** $\{C_{i},C_{j}\}\in C$ **do**:9:          $\left\{C_{i},C_{j}\right\}=KLmethod\left(C_{i},C_{j}\right)$     ▹ Employ the KL-based method to enhance intra-cluster weights and minimize inter-cluster weights.10:    **end for**11:**end for**12:$P=G^{\prime }$     ▹ Allocate clusters to the list of partitions.13:**return** *P*

The algorithm then assigns the refined clusters to the output partitions, as specified in Line 12. Ultimately, in Line 13, the SNN-GPA returns these partitions. The resulting optimal partition, when combined with a trained SNN, is then strategically mapped onto hardware grids (i.e., NoC) to facilitate efficient deployment. The NoC implementation and results are discussed in detail in Section 4.3.

## 4. Experimental Results and Discussion

### 4.1. Experimental Setup

In the preliminary phase of this study, an evaluation was conducted using a computing system equipped with a 32-core Intel Xeon Gold processor, complemented by 64 GB of RAM, and an NVIDIA Quadro P4000 GPU, operating under Ubuntu 18.04. The Python programming language was employed for the development of the tool flow. Our analysis incorporated both synthetic and realistic network models. Synthetic SNNs often involve simplified and abstract models of networks. These models capture the essential elements of spiking neurons but aim to avoid replicating the full complexity of complex NN architectures. In contrast, realistic SNNs aim to closely mimic actual NN architectures and feature extractors. This includes modeling detailed aspects of filter characteristics and modeling SNNs considering neuronal ion channels, neurotransmitter dynamics, and complex synaptic interactions. These networks are particularly useful in computational neuroscience for studying how real neural systems work. They help in forming hypotheses about neural computations and brain function.

Regarding synthetic networks, we utilized a three-layer (fully connected) synthetic network, which encompassed 4K neurons and approximately 3.75 million synapses, referred to herein as the “synthetic_4k” network. For the examination of realistic networks, a variety of models were employed, including CNN_mnist [1], LeNet_mnist [45], Zambrano_mnist [46], Rueckauer_Cifar10 [30], LeNet_cifar10 [47], AlexNet_mnist [48], multilayer perceptron for mnist (MLP_mnist), a CNN for DogsVsDogs [49], a CNN for Fruits360 [50], and AlexNet_CatsVsDogs [48] for benchmarking. The benchmarks for this evaluation were conducted using several datasets, notably the mnist handwritten digit dataset [45], the Cifar10 dataset [51], and the CatsVsDogs dataset [49]. These datasets were instrumental in evaluating the performance and efficacy of the networks under study. For the final SNN implementation, we used an existing NoC simulator (i.e., Noxim [52]).

### 4.2. Implementation Results

In the course of this research, each network was meticulously implemented using the TensorFlow framework and subsequently trained to utilize the Keras library. For the purpose of converting a CNN or ANN into a spiking neural network (SNN), we employed a modified iteration of the SNN Tool Box (SNN-TB) [30]. It is imperative to underscore the significance of weight normalization in CNNs to create accurate SNN models. A notable challenge encountered with the existing SNN-TB implementation is requiring all layer activations corresponding to the normalization dataset to be concurrently loaded into the GPU memory. This stipulation presents a considerable limitation, as large-scale models coupled with extensive datasets often exceed the memory capacity of most GPUs. To circumvent this limitation, we developed an innovative normalization workaround. While producing equivalent results, this solution reallocates the memory burden from the GPU to the system memory, thereby overcoming the previously mentioned constraint.

**Figure 2:**
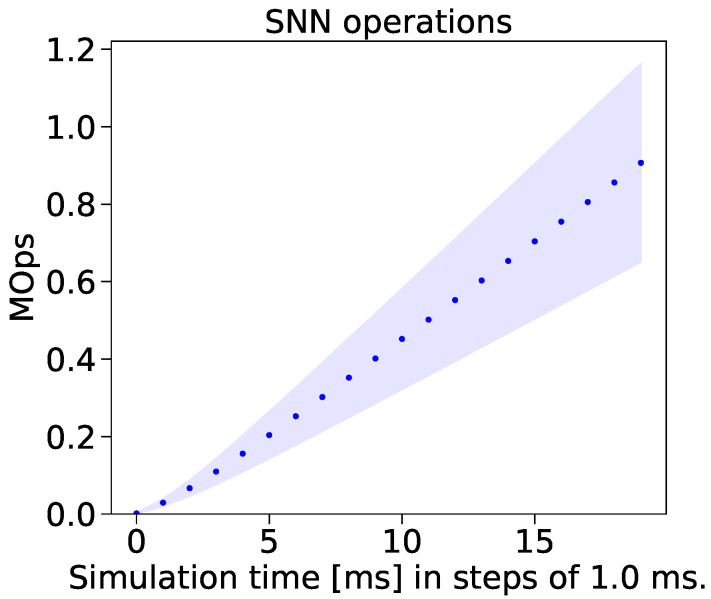
As the duration of the learning period increases, there is a notable augmentation in the activity levels of neurons. This increased activity is indicative of the dynamic nature of SNNs, where prolonged exposure to training stimuli results in enhanced neuronal responsiveness.

The firing rate of neurons in an SNN determines the amount of information being transmitted through the network. It can affect the network’s ability to learn and process information. In many SNN learning algorithms, such as spike-timing-dependent plasticity (STDP), the timing and frequency of spikes are crucial for the adjustment of synaptic weights. The firing rate thus directly influences how learning occurs in the network. At the same time, the simulation time refers to the duration for which the SNN is simulated during the training process. It is crucial for allowing the network to learn from the temporal patterns in the input data. Figure 2 shows the SNN’s million operations (MOps) versus simulation time curve considering the CNN_mnist network. We can observe increased activity in neurons as the learning time increases.

**Figure 3:**
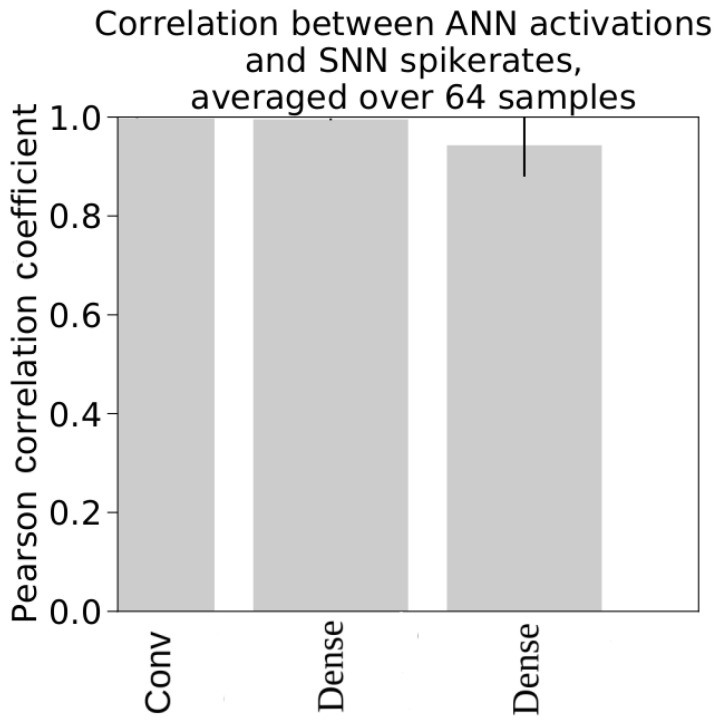
The Pearson correlation coefficients effectively substantiate the relationship between the activations of ANN layers and the spike rates of SNN layers within the CNN_mnist network framework. This statistical validation underscores the consistency and reliability of the ANN-to-SNN conversion process in this specific network context [3].

We employed Pearson correlation coefficients to ascertain the efficacy of the conversion process from ANNs to SNNs. This statistical measure was utilized to compare the activations in ANNs with the spike rates in SNNs. Figure 3 provides a graphical representation of the correlation coefficients for each layer of the CNN_mnist network [3]. This network was specifically trained using the mnist dataset, and the coefficients presented are the averages calculated over all the data batches [3].

**Figure 4:**
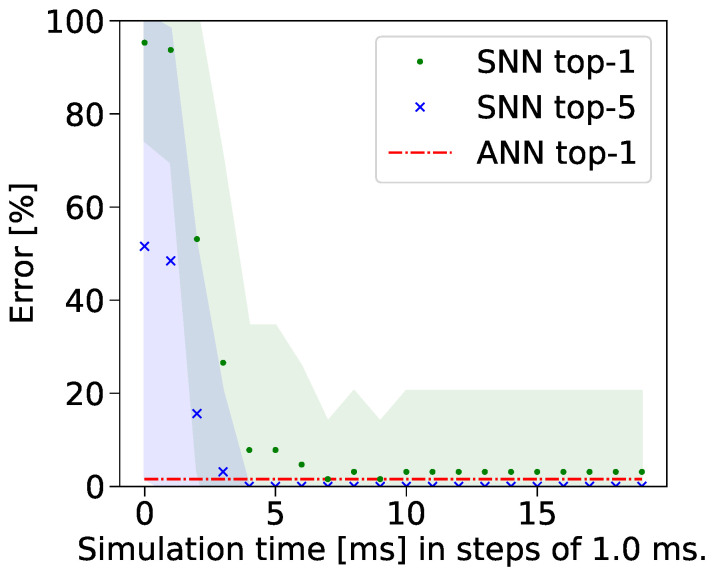
As anticipated, there is a discernible decrease in the classification error of the created SNN architecture, correlating with an augmentation in the simulation time. Conversely, the classification error associated with the ANN model exhibits a near-constant trend, which aligns with the methodology of the study, wherein a pre-trained ANN model was employed for analysis [3].

Furthermore, this study meticulously tracked the progression of classification errors throughout the training phase across various communication periods. These data are visually represented in Figure 4 [3]. The simulation duration was measured in discrete steps of 1 millisecond. In the graphical depiction, the green scatter points denote the top-1 classification errors over time, whereas the blue ones denote the top-5 classification errors observed during the same period. Additionally, the shaded regions in the figure indicate the standard deviation in the classification errors observed in both SNNs and ANNs.

Table 1 [3] presents a comparative analysis of the accuracy metrics for both the ANN and SNN models when applied to realistic networks. These networks utilized approximately 173.8 million synapses and 0.39 million neurons, on average. Furthermore, the mean spike count and the average simulation time, denoted as $S_{T}$, were recorded as 8476.14 million and 320 s, respectively. The methodology proposed herein has demonstrated its efficacy by converting ANN architectures to SNNs with a minimal average error penalty of only 2.65%.

To assess the effectiveness of our graph-partitioning methodology, we examined the synaptic weights, specifically IntraW and InterW. This analysis encompassed evaluating synthetic and realistic networks utilizing the mnist dataset. Furthermore, an SNN architecture, specifically graph_edgedet, was employed for standard edge detection, as shown in Table 2 [3]. When applied to the Zambrano_mnist network, the proposed graph-partitioning algorithm demonstrated a reduction of 6.65% in inter-communication weights and an impressive reduction of 99.86% in intra-communication weights compared to a baseline model. In an overarching evaluation, the proposed SNN-GPA achieved a reduction of 14.22% in inter-communication weights and 87.58% in intra-communication weights compared to a baseline model.

**Figure 5:**
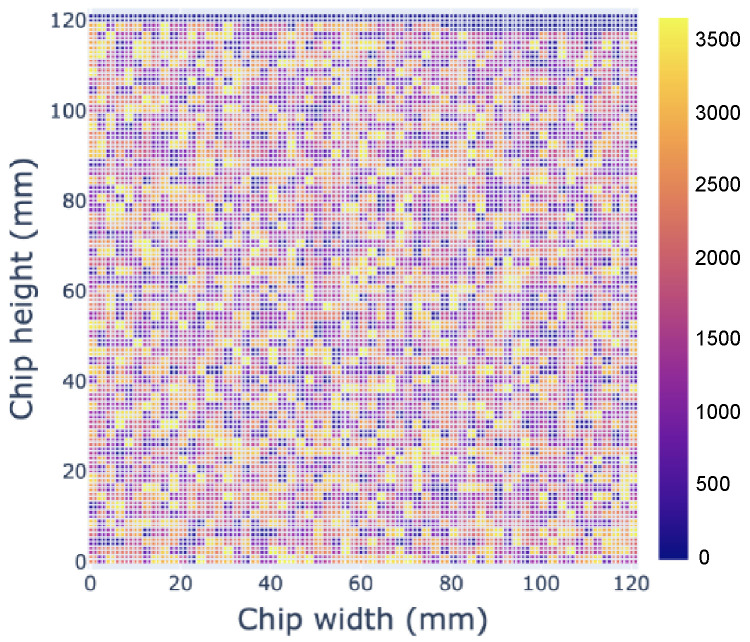
An illustrative layout depicting the placement of the Zambrano_mnist network within a 120×120 mm chip using the proposed methodology [3].

Upon completion of partition creation, the proposed methodology facilitated the placement of these neurons onto a designated NoC grid. We formulated a 2D mesh NoC architecture, adopting a grid length of 2 nm and employing a Cartesian coordinate system. Notably, this grid length is a design decision, and it can range from 10 μm to several hundred micrometers [53]. For illustrative purposes, Figure 5 presents a representative diagram depicting the placement of the Zambrano_mnist network on a 120×120 mm chip using the proposed tool flow [3].

### 4.3. Noxim NOC-Based Implementation Results

In order to understand the effectiveness of the proposed methodologies, we used the Noxim [52] simulator. The Noxim simulator was developed using SystemC, which is a library written in C++. The Noxim runtime engine (NRE) is a cycle-accurate simulator that can execute various NoC architectural features and models. Noxim supports different topologies, buffer and packet sizes, traffic distributions, routing algorithms, packet injection rates, etc. The Noxim simulator uses Tile as its primary component, which comprises a processing element (PE), local computational memory, and a router. The PE is workload-dependent and primarily responsible for consuming and generating data packets. In our analysis, we used a mesh-based NOC architecture, which has better scalability and energy efficiency compared to shared bus-based architectures—the data packet travels through the router using the existing XY algorithm.

In addition, Noxim permits a wormhole mechanism rather than the traditional store-and-forward mechanism for transferring flits from one router to another. In the wormhole mechanism, data packets are broken into smaller flits, which are then sent over the network in a wormhole fashion, whereas the conventional approach involves copying the entire data packet into the router before moving it to the next node. As a result, the wormhole approach enables resource sharing across multiple users. To demonstrate the efficacy of the proposed methodology, synthetic and realistic networks were employed, and network computations were mapped using the Noxim simulator. The results of this analysis are shown in Table 3. The synthetic_4k network exhibits the highest latency improvement of 96.88% and an energy improvement of 3.8% compared to the baseline architectures. Among the realistic networks, the latency improvement ranges from 6.27% (for LeNet_mnist_padded) to 93.83% (for Zambrano_mnist), and the energy improvement ranges from 5.66% (for Zambrano_mnist) to 56.12% (for MLP_mnist) compared to the baseline architectures, as shown in Table 3. Compared with a baseline graph-partitioning algorithm, the proposed approach demonstrates an average latency reduction of 79.74%. At the same time, the state-of-the-art SNN mapping algorithm [1] reported an average latency improvement of 45% compared to a baseline model.

**Figure 6:**
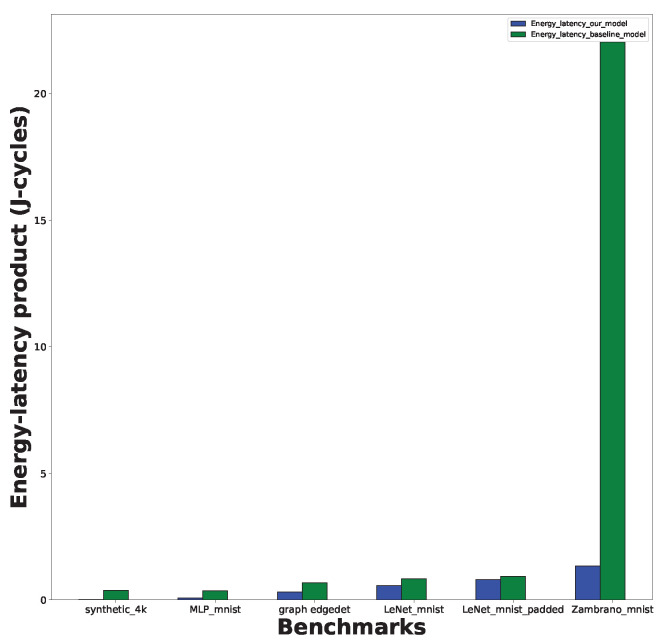
The Noxim-based implementation can lead to energy-latency product improvements of between 13.67% and 97% using the proposed graph-partitioning algorithm.

Using the proposed SNN-GPA algorithm and the Noxim tool led to a significant improvement in the energy-latency product. Figure 6 depicts the energy-latency product efficiency of the proposed algorithm. Using the proposed methodology, the synthetic synthetic_4k network exhibits a 97% reduction in the energy-latency product compared to the baseline model. Similarly, the Zambrano_mnist network exhibits a 94.17% reduction in the energy-latency product compared to the baseline model, with the highest energy-latency efficiency among the realistic networks. On average, the networks listed in Table 3 exhibit an 82.71% reduction in the energy-latency product compared to the baseline architectures.

## 5. Conclusions

This paper introduces a comprehensive tool flow designed for the exploration and implementation of high-level NN architectures, mainly focusing on SNN models. This tool flow integrates the use of Python Keras libraries and the SNN-TB, along with our innovatively developed SNN-GPA. The SNN-GPA is instrumental in partitioning and positioning the SNN within an NoC architecture. The methodology proposed herein demonstrates remarkable efficiency in converting ANN architectures into SNNs, incurring an average error penalty of only 2.65%. Moreover, in comparison to a baseline model, the SNN-GPA significantly reduces synaptic communication weights, with an average reduction of 14.22% in inter-communication weights and 87.58% in intra-communication weights. This underscores the efficacy of the proposed algorithm in optimizing neural network communication pathways and emphasizes the effectiveness of the proposed approach in enhancing the operational efficiency of SNN models. In contrast to a baseline graph-partitioning algorithm, the suggested approach demonstrates an average latency reduction of 79.74% and a decrease in energy consumption of 14.67%. Using the proposed methodology, the synthetic synthetic_4k network exhibits a 97% reduction and the realistic Zambrano_mnist network exhibits a 94.17% reduction in the energy-latency product compared to the baseline model.

## Figures and Tables

**Table 1:** The average quantities of synapses and neurons within the network are quantified at 173.8 million and 0.39 million, respectively. The methodology developed in this study proficiently facilitates the conversion of ANNs to SNNs, particularly when applied to realistic benchmark scenarios. This conversion is achieved with an impressively low average error penalty, recorded at merely 2.65% [3].

Application	# of Synapses (M)	# of Neurons (M)	# of Spikes (M)	$S_{T}$ (s)	SNN Acc. (%)	ANN Acc. (%)
CNN_mnist [1]	1.61	0.01	79.33	200	97.947	98.46
LeNet_mnist [45]	0.29	0.007	93.72	200	98.037	98.88
Zambrano_mnist [46]	1.42	0.01	125.10	200	99.25	99.36
Rueckauer_Cifar10 [30]	2.50	0.11	7786.46	1000	79.43	81.25
LeNet_Cifar10 [47]	0.66	0.01	752.26	200	53.596	60.64
AlexNet_mnist [48]	923.45	0.79	9550.44	500	97.24	98.54
MLP_mnist	0.20	0.001	30.27	200	97.33	97.60
CNN_CatsVsDogs [49]	522.82	2.03	9290.56	50	91.60	93.88
CNN_Fruits360 [50]	96.09	0.40	48,092.77	600	89.5	96.71
AlexNet_CatsVsDogs [48]	165.01	0.53	8960.47	50	77.7	82.78
**Average**	173.80	0.39	8476.14	320	88.16	90.81

**Table 2:** The SNN-GPA introduced in this research is applied to both synthetic and realistic network models. Comparative analysis with a baseline graph-partitioning approach reveals that the proposed SNN-GPA yields a significant reduction in synaptic communication. On average, there is a 14.22% decrease in inter-synaptic communication and an 87.58% reduction in intra-synaptic communication, underscoring the effectiveness of the proposed algorithm in optimizing NN communication pathways [3].

Metric	Synthetic_4k	MLP_mnist	Graph_edgedet	LeNet_mnist	LeNet_mnist_padded	Zambrano_mnist
# of nodes	4000	1050	4377	6598	9118	14,554
# of clusters	250	66	274	413	570	910
$ of edges	3,750,000	203,264	54,876	286,120	422,824	1,422,848
InterW (base)	536,702.65	918.05	5,332,848.19	5236.54	5885.53	53,811.83
IntraW (base)	1877.17	12.05	4421.91	9.59	10.45	37.37
InterW (our)	535,628.61	465.10	4,322,871.32	4967.05	5588.75	50,235.91
IntraW (our)	2951.21	465.05	1,014,398.78	279.08	307.24	3613.29
Inter (%)	0.20	49.34	18.94	5.15	5.04	6.65
Intra (%)	36.39	97.41	99.56	96.56	96.60	98.97
Runtime (s)	373.16	3.06	53.51	235.80	371.46	1113.34
**Average improvement**	**Inter: 14.22%; Intra: 87.58%**					

**Table 3:** Leveraging Noxim, we conducted mapping exercises for both synthetic and realistic networks. In comparison with a baseline graph-partitioning algorithm, the proposed approach demonstrates an average reduction of 79.74% in latency and 14.67% in energy consumption.

Benchmark	Grid Size	# of Inter-Flits (Baseline)	# of Inter-Flits (Ours)	Latency (Cycles)			Energy (μJ)		
				**Baseline**	**Ours**	**Improvement (%)**	**Baseline**	**Ours**	**Improvement (%)**
synthetic_4k	16×16	145,081	144,791	9451.92	295	96.88	38.88	37.40	3.80
MLP_mnist	16×8	160,912	81,521	14,354.03	7099	50.54	25.07	11.00	56.12
graph_edgedet	32×16	191,623	155,332	11,895.95	7567	36.39	56.25	41.70	25.87
LeNet_mnist	32×16	204,164	193,657	10,382.31	9170	11.68	80.55	62.10	22.90
LeNet_mnist_padded	32×32	231,106	219,452	10,319.36	9672	6.27	89.89	82.80	7.89
Zambrano_mnist	32×32	4,383,696	294,351	158,831.19	9806	93.83	144.37	136.20	5.66
**Average**		**886,097.11**	**181,517.33**	**35,872.46**	**7268.17**	**79.74**	**72.50**	**61.87**	**14.67**

## Data Availability

Data are contained within the article.
